# The bacteremia of dental origin and its implications in the appearance of bacterial endocarditis

**DOI:** 10.4317/medoral.19562

**Published:** 2013-10-13

**Authors:** María R. Mang-de la Rosa, Lizett Castellanos-Cosano, María J. Romero-Perez, Antonio Cutando

**Affiliations:** 1 Department of Special Care patients, School of Dentistry, University of Granada, Granada, Spain; 2 Associate Professor of the Master Program of Dentistry for Special Patients. School of Dentistry. University of Seville. Seville. Spain; 3 Department of Special Care patients, School of Dentistry, University of Granada, Granada, Spain; 4 MD, DDS, PhD, Special Care Professor, University of Granada, Department of Special Care in Dentistry, School of Dentistry, University of Granada, Granada, Spain

## Abstract

Numerous systemic diseases may affect the oral cavity and vice versa,in particular severe diseases that involve the heart valve. In these cases, additional measures or a modification to our dental treatment need to be taken. We are aware of various diseases that can cause the emergence of bacterial endocarditis (BE), such as; rheumatic fever, valve lesions due to intravenous drug use, Kawasaki disease and valve surgery, among others. Due to its severity when it is not taken into account in dental treatment, we intend to show the evolution of the antimicrobial prophylaxis towards this condition. Furthermore, we intend to publish the current guidelines of institutions and societies which increasingly encourage rational antimicrobial use. In addition, we intend to examine the evidence of the possible origins of this disease during dental treatment and at the same time describe the necessary considerations that need to be taken during dental treatment.

** Key words:**Endocarditis, antibiotic profilaxis, dental treatment.

## Introduction

- Rheumatic fever

* Concept:

Rheumatic fever is an inflammatory disease that can appear after an initial phase of pharyngitis. It is associated with certain strains of beta-hemolytic streptococci (Streptococcus pyogenes).

* Epidemiology:

It currently has a low incidence in western populations. However, it is still common in other regions of the world such as the Indian subcontinent, the Middle East and some Caribbean islands, regions from which we receive immigrants.

As far as age is concerned, the peak age of incidence is between 5 to 15.

* Clinical profile:

After the initial symptoms of pharyngitis a spike of fever may occur within weeks. It can be accompanied by migratory arthralgia. This process usually disappears within 6 to 12 weeks without apparent side effects. During the acute phase of the disease it is possible to detect small changes in the heart function, leading in rare cases to heart failure and death.

After this process, in some cases the patient can develop a chronic rheumatic heart disease causing permanent damage to the heart. It is seen essentially as a mechanical and hemodynamic condition, in which the defective valves can become infected resulting in bacterial endocarditis (BE).

* Diagnosis:

Clinical expression of rheumatic fever is so variable that it can only be diagnosed if at least two of the following major criteria are met ( Table 1).

**Table 1:**
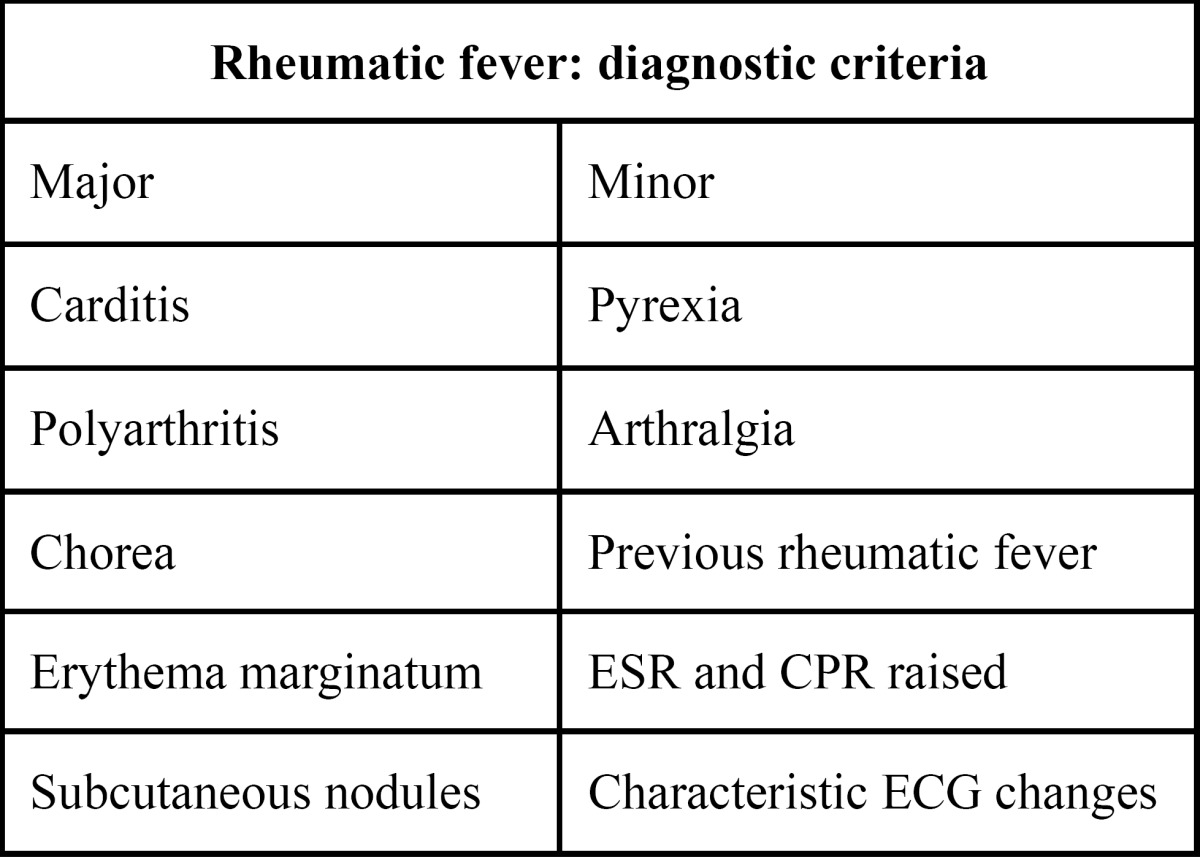
Rheumatic fever: diagnostic criteria.

* Further Examinations:

Low titers of antistreptolysin O (ASO) antibodies rule out a strep infection. High titers suggest it, however it is not a diagnosis of the disease.

* Prognosis:

Approximately, 60% of children who had survived the acute phase of rheumatic fever in the past developed heart damage at the age of 10, although they later experienced heart failure (1). Nevertheless, these complications are rare in developed countries.

The mitral valve is most frequently affected. An early symptom of damage to the valve is heart murmur. Other possible complications include stenosis, valve insufficiency or heart enlargement. These complications can be detected radiographically. Additionally, they can be revealed through ECG or echocardiographic changes. Subendothelial inflammation is the most serious complication.

* Treatment:

Streptococcal pharyngitis generally requires antibiotic penicillin treatment. An early antimicrobial treatment in less than 24 hours prevents further rheumatic fever development in most cases. Due to risks of recurrence (phenoxymethylpenicillin, or sulfonamide in case of allergies) constant antibiotic prophylaxis is needed to reduce the risk of a permanent heart disease. We should take into account that most of the patients suffering from a permanent feature of this disease might present an anticoagulant state.

## Kawasaki disease (Mucocutaneous lymph node syndrome)

Kawasaki disease is an acute and febrile disease which could present lymphadenopathy and desquamation of lips and fingers. It can also simulate scarlet fever and erythema multiforme. Nowadays, much more importance is given to this syndrome as potentially causing a severe heart disease in children, than to rheumatic fever. The disease shows a particular prevalence in the male population (2:1). Additionally, 80% affected people were under the age of 5 (2).

Among the possible etiological pattern we presume an infectious origin, possibly related to a specific genetic factor. Nonetheless, no agents have been isolated up to now.

The main disease process presents with vasculitis throughout the body. This process could unleash a coronary aneurysm. The duration of fever which normally appears during the disease is normally no less than 5 days, and erythema and edema might occur on body extremities followed by desquamation, conjunctivitis, strawberry tongue and cervical lymphadenopathy. The death rate is around 5-10 % and due basically to an acute myocardial infarction (AMI) caused by a previous thrombotic aneurysm.

Regarding the treatment, patients should be admitted to hospital to undergo a Gamma globulin treatment. Other possible prescriptions might be aspirin or systemic steroids.

## Valve lesions induced by drugs

A combined consumption of appetite suppressants, as i.e. fenfluramine and phentermine, has been described as a risk factor in developing cardiac valve regurgitation, especially in relation to the aortic valve (3). Patients who might have consumed these kinds of drugs for more than 3 months should undergo a clinical checkup. In case of any abnormality, the patient should be examined by a cardiologist and given an echocardiography.

## Heart valve surgery

Heart valve dysfunctions might be repaired by a valvotomy, grafts or valve prosthesis.

Patients undergoing a cardiac surgery should have optimal oral hygiene conditions prior to the surgery. Generally, teeth presenting a bad pulp or periodontal prognosis should be removed, especially if considering a valve replacement, a major surgery related to congenital abnormalities or a heart transplant. Teeth with superficial caries and periodontal pockets should be preserved.

After a heart surgery unnecessary dental treatments should be avoided at least for the first 6 months due to the fact that many patients might be undergoing an anticoagulant, immunosuppressive treatment, following medical prescriptions or presenting any other residual lesion.

Heart valve prosthesis is particularly prone to bacterial endocarditis which might be related to a higher death rate. Bacterial endocarditis within the first 6 months is normally caused by Staphylococcus aureus. It rarely has a dental origin presenting an average mortality rate of 60% (4).

## Infective endocarditis

* Concept:

Bacterial endocarditis (BE) is a disease caused by a bacteremia that affects different organs or tissues, including the oral cavity. Although it has a low incidence, it might imply a potential threat to the life of the affected individual. Predominantly it tends to develop on cardiac valves previously damaged, the mitral valve being its most frequent location, followed by the aortic and in rare occasions the pulmonary valve.

* Epidemiology:

The incidence is estimated to be 1 to 5 cases per 100.000 inhabitants (5). It is rare amongst younger population with the exception of intravenous drug users.

Heart valves, sometimes damaged by diseases (i.e. a rheumatic heart disease) may frequently be affected by bacterial endocarditis (BE), although this disease might equally affect those people suffering from a congenital heart disease or after undergoing a valve surgery. Conversely, those people affected by a non-complex myocardial infarction or having faced non-complex angioplasties, coronary bypass or cardiac pacemakers are not likely to risk a bacterial endocarditis (BE) infection ( Table 2).

**Table 2:**
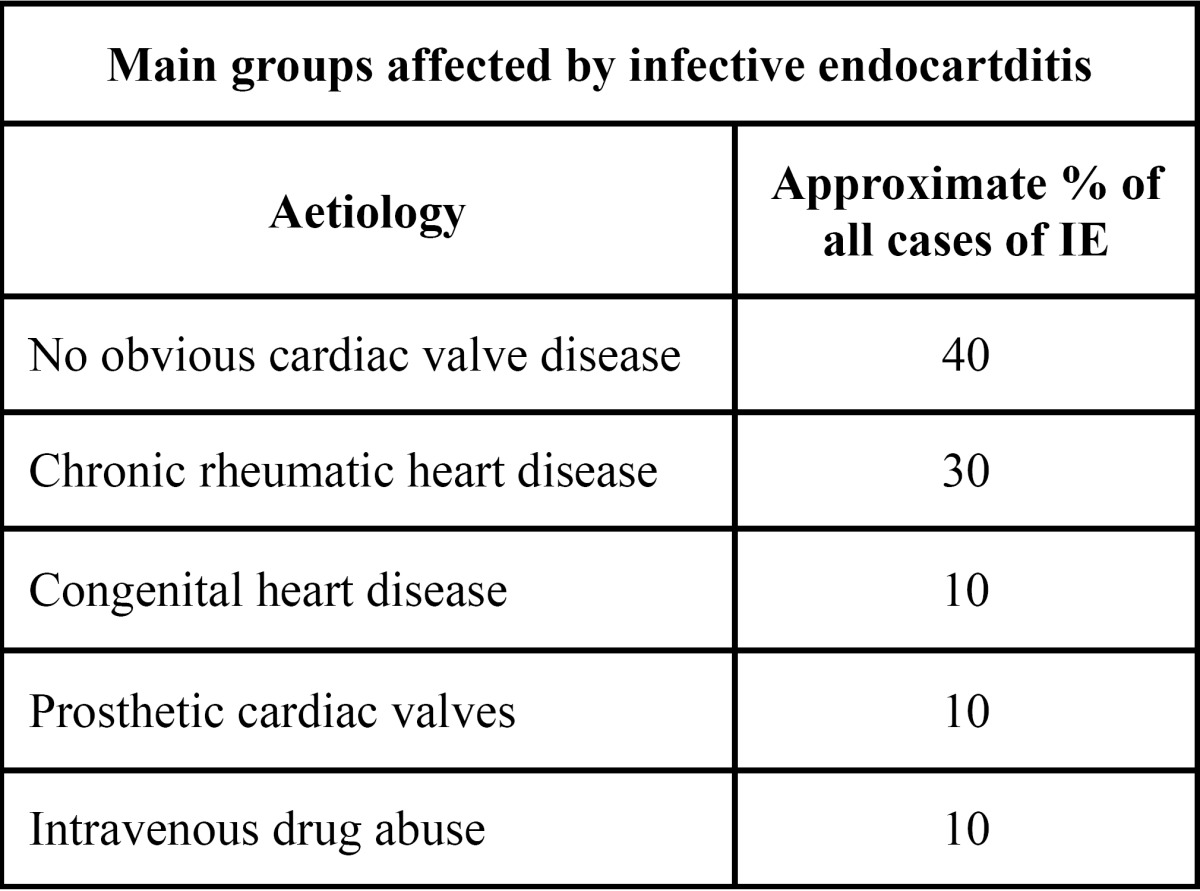
Relationship between IE and risks groups.

It is noteworthy that cardiac valves previously affected by a bacterial endocarditis or those relying on a prosthesis are especially prone to suffer from this infection ( Table 1)

* Origin and causes of the disease:

Typically the bacterial endocarditis is caused by adenoids, composed by platelets and fibrin, which although originally considered sterile might be colonized by microorganisms. Most of the bacteremias are momentary, self-restrictive and not associated to any other systemic complication.

Factors involved in the development of a bacterial endocarditis are difficult to define but a vulnerable surface (i.e. a damaged endocardium) and a high bacterial load in the blood seem to be decisive.

Causing microorganisms in 90% of the cases are staphylococcus, streptococcus and enterococcus. Oral streptococci belong to viridans group (streptococcus mutans and streptococcus sanguis). Being part of dental plaque they might enter the bloodstream causing bacteremia through daily habits like chewing or tooth brushing. Dental extraction or other dental procedures might cause bacteremia as well.

As a result of dental treatment a small amount of patients contract bacterial endocarditis (EB). Developing bacterial endocarditis (EB) in valve diseases patients is statistically 1 every 3000 cases.

Intravascular access might be often infected; however, there is no evidence of a dental origin for these infections. Furthermore, the abuse of intravenous drugs may end up in bacterial endocarditis (EB). In this case bacteria penetrate through the needle.

* Clinical profile:

Bacterial endocarditis cases vary from a fulminant and acute attack to a chronic evolution. Light pigmentation of the skin, joint pains or hepatosplenomegaly is typical; however, the main effect of endocarditis is heart damage (valve destruction and heart failure). The progressive sign of disability is related to changing murmurs showing heart damage, infection or embolic damage in various organs, especially in the kidneys. The liberation of emboli may have general effects from a loss of the peripheral pulse to sudden death due to stroke.

* Diagnosis:

The diagnosis of bacterial endocarditis is based on four factors: changing murmurs, ECG diagnosing abnormal rhythms of the heart, echocardiography identifying adenoids and evaluating valve and heart functions and blood culture. It is essential to perform blood culture before antibiotic treatment begins, at half-hourly intervals in order to increase the prospects of positive blood cultures.

* Prognosis and treatment:

Without treatment, bacterial endocarditis is a fatal disease in 30% of the cases. Patient should be sent to hospital for intravenous antibiotic therapy (benzylpenicillin and gentamicin are normally used). Generally an extended treatment is needed. For this reason, programs of home hospitalization service have been progressively adopted administering antibiotics intravenously. If staphylococcal endocarditis is suspected, penicillin could be replaced for vancomycin. In severe cases like prosthetic valve endocarditis patients it might be necessary to replace the infected valve with a new valve.

* Dental considerations:

It is essential to promote good oral health in patients at risk of endocarditis. This is the best way to reduce the necessity of surgery in these patients. Nevertheless, this aspect of dental treatment is often neglected and a high percentage of patients in cardiology clinic suffer from a periodontal disease. Additionally, there is no reliable evidence to suggest that oral hygiene methods such as electric toothbrushes, irrigators or other similar devices may be a health risk.

In many countries there are national guidelines in order to use antimicrobial prophylaxis having a bacterial endocarditis condition, in case a dental surgery is needed. The reduction of bacteremia, preventing the adherence of bacteria to the endocardium (6), is the main benefit of the use of prophylaxis.

Nonetheless, the effectiveness of these guidelines have been questioned by several authors, publications and societies, such as; the recommendations published in France in 2002, the British Society for Antimicrobial Chemotherapy (BSAC) in England (2006), the American Heart Association (AHA) in 2007 and the National Institute for Health and Clinical Excellence (NICE) in the UK in 2008. Finally, these guidelines have been questioned also by australian recommendations in 2008 and the European Society of Cardiology (ESC) in 2009.

In relation to antibiotic prophylaxis in bacterial endocarditis (BE), medico-legal services carefully advice to dentists.

- Full discussion with all patients

- Ask for medical advice in case of doubt

- Administering antibiotic prophylaxis to risk patients in countries where national guidelines for antibiotic prophylaxis are provided. For instance, the American Dental Association (ADA), the Infectious Diseases Society of America (IDSA) and the Pediatric Infectious Diseases Society (PIDS)recommended to use antibiotic prophylaxis in dental procedures but only for patients in high-risk situations, such as those having.

- A prior history of infective endocarditis (IE)

- A prosthetic heart valve

- Congenital heart abnormalities, especially:

a) An unrepaired cyanotic congenital heart disease, including those with palliative shunts.

b) A completely repaired congenital heart defect with prosthetic material or device, whether placed by surgery or by catheter intervention. During the first six months after the procedure, prophylaxis is recommended because endothelialization of prosthetic material occurs within this time.

c) Any repaired congenital heart defect with residual defect at the site or adjacent to the site of a prosthetic patch or a prosthetic device (7).

d) A cardiac transplant that develops a problem in a heart valve.

Similarly, the American Heart Association (AHA) stated that in gastrointestinal and genitourinary procedures there is no longer need to use antibiotic prophylaxis.

Considering the special conditions of aboriginal australians, australian recommendations corrected AHA recommendations.

The European Society of Cardiology (ESC), endorsed by the AHA, recommended antibiotic prophylaxis use only for high risk patients of IE and in dental treatments involving manipulation of the periapical region or gingival tissue or perforation of the oral mucosa.

Through the years, in the UK have been emerging societies covering endocarditis. The British Society for Antimicrobial Chemotherapy (BSAC) recommended in 1992 the covering for all the procedures related to bleeding.

The British Cardiac Society and the Royal College of Physician (BCS/RCP) recommended in 2004 antibiotic prophylaxis for all dental procedures that might cause a bacteremia, as for those related to heart defects and heart surgery. The British Society for Antimicrobial Chemotherapy (BSAC) recommended in 2006, endorsing the recommendations made in France, antibiotic prophylaxis use only for high risk patients of BE in cases which these patients were at high risk of death.

In the UK over the years, many schemes have suggested coverage for endocarditis. The British Society for Antimicrobial Therapy (BSAC) in 1992 recommended for the coverage of all procedures associated with bleeding. The British Cardiac Society and the Royal College of Physicians (BCS / RCP) in 2004 suggested antibiotic prophylaxis for all dental procedures that may trigger a bacteremia and for a large number of defects and / or cardiac surgery. In 2006, in line with the recommendations made in France, the BSAC recommended antibiotic prophylaxis only in patients at high risk for EB, and should develop it, have a high risk of death. These cases include:

- A prior history of infective endocarditis (IE)

- A prosthetic heart valve

- Pulmonary shunts or surgically constructed conduits.

Additionally, prophylaxis was limited to dental treatments including those involving dentogingival manipulation.

Shortly after, in 2008, the National Institute for Health and Clinical Excellence (NICE) recommended that antibiotic prophylaxis use was no longer needed in dental practice.

Antibiotic prophylaxis is not recommended in patients at risk of endocarditis having a dental treatment. The National Institute for Health and Clinical Excellence (NICE) issued a list including heart situations at high risk of suffering an infective endocarditis (IE). It was issued in order to increase vigilance in these patients as well as for highlighting the importance of maintaining good oral health for reducing the need of surgery.

It is essential that the odontologist is aware of current guidelines related to prophylaxis for bacterial endocarditis (BE). Tomás and cols. 2002 study shows a telephone survey of 400 Spanish professionals in which only a 30,9% of the surveyed worked in agreement with AHA and BSAC guidelines (8).

Therefore, as it has been previously said, national and international societies have recently published new guidelines in order to prevent bacterial endocarditis. These societies encourage rational antimicrobial use and condemn the indiscriminate use of antibiotic prophylaxis for dental procedures ( Table 2).

Among the reasons that support or restrict prophylaxis it is included an antibiotic-related adverse effect like anaphylactic shock. These reactions are estimated to occur in 5% of patients, being equally the risk in oral penicillin and in intramuscular penicillin.

2.9 % of antibiotic-related adverse effects are a result of amoxicillin use, including anaphylactic reactions, skin reactions, gastrointestinal disorders, liver problems and hematological complications (9-11). The higher the indiscriminate use of antibiotics is, the greater the possibility that the risk of adverse reactions may exceed the risk of infective endocarditis (IE). Estimations suggest that deaths from anaphylactic reactions to antibiotics may be five to ten times more common than deaths from infective endocarditis (IE). Studies show that hypersensitivity reactions to penicillin or anaphylaxis occur from 0.04 to 0.11% of the cases in which patients were given penicillin, being the risk higher intravenously (12).

Bor et al. (13) study estimates that among 10 million patients with mitral valve prolapse where no antimicrobial prophylaxis was given, 47 cases of bacterial endocarditis would occur. Additionally the study says that 2 fatal cases of bacterial endocarditis would occur. If all these patients were given prophylaxis with a penicillin the cases of bacterial endocarditis would be reduced to 5 and the endocarditis would not cause death; nonetheless, 175 deaths due to penicillin reactions would occur.

Considering that antibiotic use in dentistry may be up to 10% of the total antibiotic use, we have to take into account the risk of developing bacterial resistance (14-16). If a antimicrobial prophylaxis is provided, many dental treatments should be performed to reduce the episodes of needed antibiotic (16). If several prophylaxis episodes are required, they should be spaced two weeks at least. Treatment should be stopped 3 to 4 days in case the patient is having other antibiotics (17,18).

As far as Oliver et al. study (19) is concern, there is no scientific evidence demonstrating that antibiotic prophylaxis is effective to prevent infective endocarditis. Moreover, the absence of large-scale clinical trial publications demonstrating the effectiveness of prophylaxis is a fact. For this reason, these guidelines have been questioned.

Several epidemiological studies have estimated that 14% to 20% of cases of bacterial endocarditis (EB) are related possibly to oral hygiene origin (20-22). However, dental treatment is a rare cause of bacterial endocarditis (BE), being toothbrushing the most frequent source of bacteremia.

A study conducted by Strom et al. (23) did not observe in the previous 3 months to the diagnosis an increased frequency of dental treatment in bacterial endocarditis patients. The study concluded that only few cases of endocarditis would have been prevented if antibiotic prophylaxis were given in dental treatments being a 100% rated its effectiveness. Only a small fraction of cases (5.3%) would have been potentially prevented if antibiotic therapy were a 100% effective and given to all patients at risk in dental treatment, as another study concluded.

Nowadays, there is a solid scientific evidence that bacteremias by microorganisms causing endocarditis occur in routine daily events, such as tooth brushing. A study by Hupp et al. estimated in fact that brushing a child’s teeth twice a day would cause 154,000 times more risk to bacteremias than a single tooth extraction (24). It is also estimated that there is a risk 5.6 million times bigger of suffering from a bacteremia by toothbrushing than by a single dental tooth extraction (25).

Similarly, other authors suggest that daily events, such as chewing or flossing can cause bacteremia of an oral origin. Microorganisms enter the bloodstream in these events as a result of small movements of the tooth in the alveolus. These movements originate intermittent positive and negative pressures in the alveolus resulting in microscopic valvular lesions.

Thus, Lockhart et al. (26) conducted a double-blind randomized controlled clinical trial in which it was demonstrated a relation of bacteremia to bad hygiene as a result of tooth brushing. Furthermore, it was also determined a relation of bacteremia to gingival bleeding after tooth brushing. These results suggest that would be more effective to promote oral hygiene and eliminate gingivitis. Additionally, the incidence of bacteremia after tooth brushing would reduce, as well as the need for dental extraction. Thus, bacteremias in these events would also reduce ( Table 3).

**Table 3:**
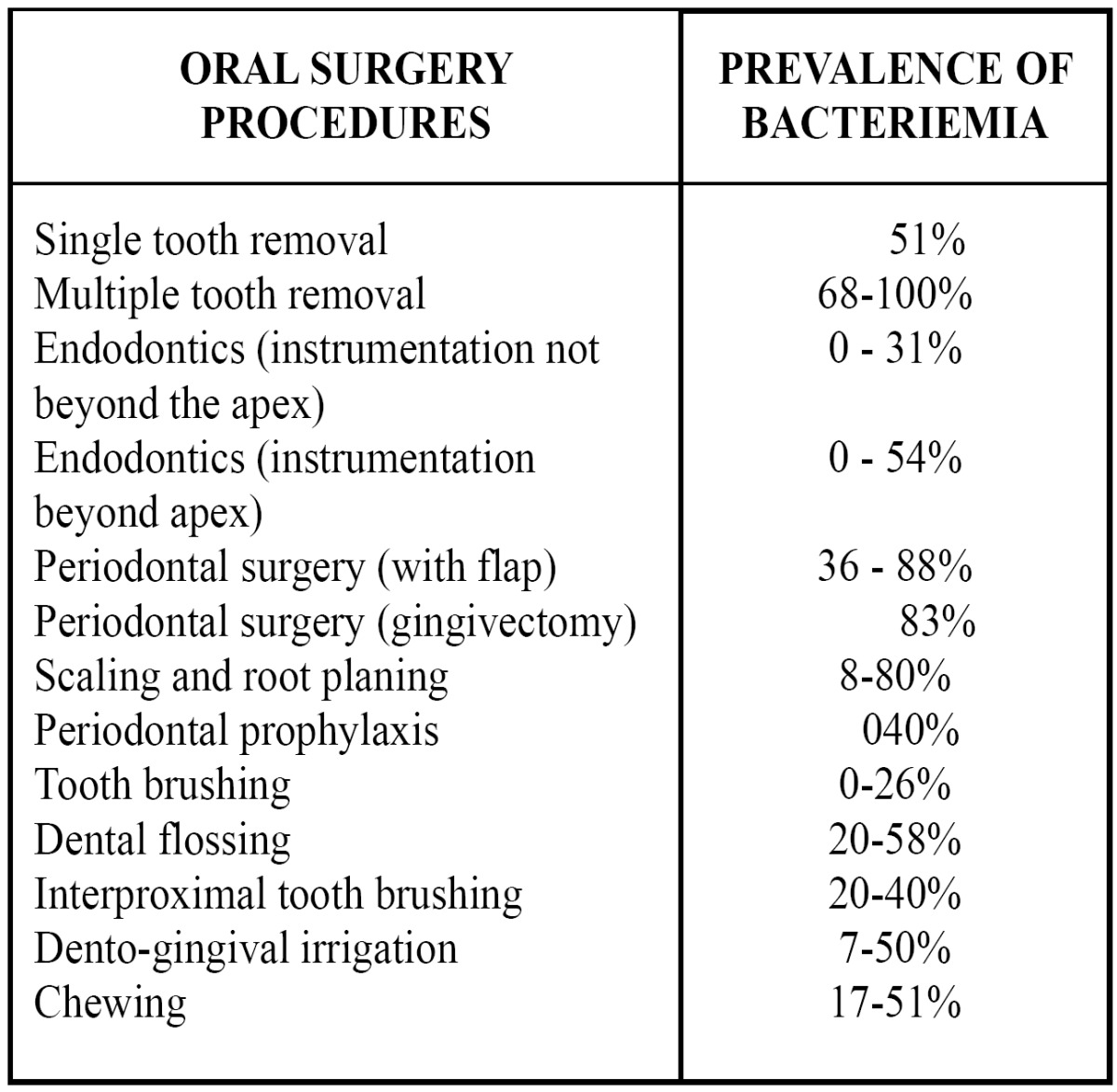
Relationship between bacteriemia and oral procedures.

Bacteremia might be also the result of an invasive procedure. In dental infections risk increases. It has been estimated that from 8 to 10% of endocarditis are related to oral infections with no oral bleeding treatment (11). This is due to the permeability of the epithelium that surrounds the tooth-gingival tissue interface and the prostaglandins in blood that increase the number of leukocytes and fibrinogen. As a consequence, the speed of the blood circulation will reduce and bacteria may enter (27). In these cases, a synergistic effect between periodontal or periapical condition and the invasive bacteria would contribute to develop later a bacterial endocarditis (BE). After bleeding dental procedures, bacteremia tends to last less than 15 minutes (12).

It has been shown that the number of colonies isolated from oral origin is low, if we compare it to the inoculum needed to induce a bacterial endocarditis (BE) in experimental animal studies (28).

Piñeiro et al. (29) study suggests that there is no significant risk of bacteremia in implats. Therefore they rule out a prophylaxis use in these cases. Finally,in line with BSAC recommendations, the study promotes chlorhexidine mouthrinse (0.2%) before dental implant surgery.

Hupp et al. (24) Farbod et al. (30), study questions that dental procedures may cause bacteremia. As far as Porat Ben-Amy et al. (31) study is concerned, it demonstrated that dental procedures do not increase the risk of suffering from infective endocarditis (IE). Therefore, there is no need for prophylaxis.

It is necessary, because of discrepancies between different authors and studies, to conduct prospective double-blind randomized controlled studies. Nevertheless, these studies are not conducted by ethical reasons.

The use of antiseptic mouthwash has been demonstrated to significantly reduce the risk of oral origin bacteremia. As a result, a 0.12% chlorhexidine rinse for 30 seconds was chosen (32,33).

In 1945, was stated that the practice of dental extractions led to the entrance of bacteria into the bloodstream due to the rupture of blood vessels in the gingival sulcus and the pumping effect induced by the manipulation (34).

The evidence regarding prevalence between surgical and non-surgical procedures are shown in  Table 4. An elevated prevalence it has been observed in post-extraction blood cultures when compared with extraction of impacted or partially erupted third molars and after more aggressive maxillofacial surgical techniques. Recently, had been showed that even implant placement via a mucoperiosteal flap does not carry a significant risk of producing bacteraemia compared with the baseline percentage (29) ( Table 4). A possible explanation for these results is that the periodontal space is not invaded in these other surgical procedures, a factor which would suggest that this space represents the critical region from which oral bacteria enter the bloodstream (35). When evaluating non-surgical dental interventions, the prevalence of bacteraemia was similar after conservative dental procedures and after other orthodontic procedures and was lower after performing root canal treatment (0%-42%) ( Table 4).

**Table 4:**
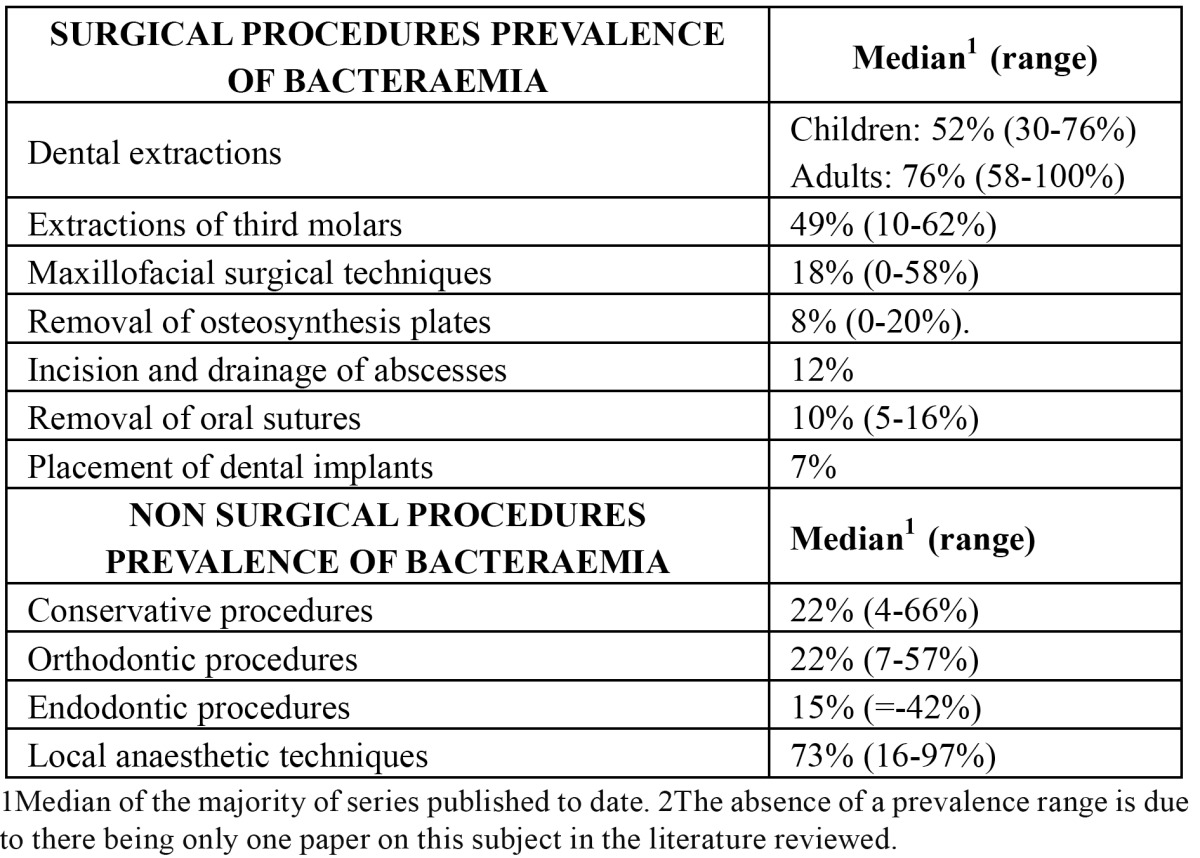
Prevalence of bacteraemia following surgical and non-surgical dental manipulations.
